# Glioblastoma and glioblastoma stem cells are dependent on functional MTH1

**DOI:** 10.18632/oncotarget.19404

**Published:** 2017-07-20

**Authors:** Linda Pudelko, Pegah Rouhi, Kumar Sanjiv, Helge Gad, Christina Kalderén, Andreas Höglund, Massimo Squatrito, Alberto J. Schuhmacher, Steven Edwards, Daniel Hägerstrand, Ulrika Warpman Berglund, Thomas Helleday, Lars Bräutigam

**Affiliations:** ^1^ Department of Medical Biochemistry and Biophysics, Science for Life Laboratory, Division of Translational Medicine and Chemical Biology, Karolinska Institutet, Stockholm, Sweden; ^2^ Cancer Cell Biology Programme, Seve Ballesteros Foundation Brain Tumor Group, Centro Nacional de Investigaciones Oncológicas, CNIO, Madrid, Spain; ^3^ Department of Applied Physics, Science for Life Laboratory, Royal Institute of Technology, Stockholm, Sweden; ^4^ Department of Oncology-Pathology, Karolinska Institutet, Stockholm, Sweden; ^5^ Department of Oncology, Lab of Tumor Inflammation and Angiogenesis, KU Leuven, Leuven, Belgium

**Keywords:** MTH1, Nudt1, DNA damage, glioblastoma multiforme, cancer stem cells

## Abstract

Glioblastoma multiforme (GBM) is an aggressive form of brain cancer with poor prognosis. Cancer cells are characterized by a specific redox environment that adjusts metabolism to its specific needs and allows the tumor to grow and metastasize. As a consequence, cancer cells and especially GBM cells suffer from elevated oxidative pressure which requires antioxidant-defense and other sanitation enzymes to be upregulated. MTH1, which degrades oxidized nucleotides, is one of these defense enzymes and represents a promising cancer target. We found MTH1 expression levels elevated and correlated with GBM aggressiveness and discovered that siRNA knock-down or inhibition of MTH1 with small molecules efficiently reduced viability of patient-derived GBM cultures. The effect of MTH1 loss on GBM viability was likely mediated through incorporation of oxidized nucleotides and subsequent DNA damage. We revealed that MTH1 inhibition targets GBM independent of aggressiveness as well as potently kills putative GBM stem cells *in vitro*. We used an orthotopic zebrafish model to confirm our results *in vivo* and light-sheet microscopy to follow the effect of MTH1 inhibition in GBM in real time.

In conclusion, MTH1 represents a promising target for GBM therapy and MTH1 inhibitors may also be effective in patients that suffer from recurring disease.

## INTRODUCTION

Malignant glioblastoma multiforme (GBM) represents the most prevalent form of brain tumors and despite the combinatory treatment with radiation with Temozolomide, the prognosis of glioma patients is dismissive as inherent or acquired resistance lead almost inevitably to tumor recurrence and patient death within barely 15 months. The current consensus is that the recurring tumor develops from treatment-resistant GBM stem cells (GSC) [1], and since radiotherapy could not be substantially improved in the last years, novel chemotherapeutic agents that are also targeting GSCs are of urgent need [2].

Cancer cells are characterized by a specific redox environment that adjusts the oncogenic metabolism to meet the specific demands of the tumor cell, and allows the cancer to grow and metastasize [3]. The cancer redox environment is, however, accompanied by a high-oxidative burden that requires upregulation of antioxidant enzymes and other non-oncogenic addiction enzymes [4]. It has been reported that the free nucleotide pool is especially prone to oxidative damage [5], which in consequence could lead to nucleotide mispairing, mutations and cell death [6] [7]. Among the nucleotides, dGTP is most prevalently oxidized and the product, 8-oxo-dGTP, can induce mutagenic transversions. There is a large body of evidence that the human mutT homologue (MTH1), which hydrolyzes 8-oxo-dGTP, represents an essential non-oncogenic addition enzyme [8][9], and we and others have shown that MTH1 is a potent anti-cancer target [10][11]. Moreover, our group has developed specific small molecule inhibitors including TH588 [10] and TH1579 [12] that target MTH1 and possess promising anti-cancer activity *in vitro* and *in vivo* [10]. Besides representing a general phenomenon in cancer cells, high oxidative pressure has also been reported in brain cancers including GBM [13-15]. Since recent data from others and our lab underlines the important link between the cellular redox environment and dependency of functional MTH1 [16, 17], we sought to investigate if the oxidative pressure of GBM cells renders them vulnerable to MTH1 inhibitors.

## RESULTS

### MTH1 is upregulated in Glioblastoma multiforme

**Figure 1:**
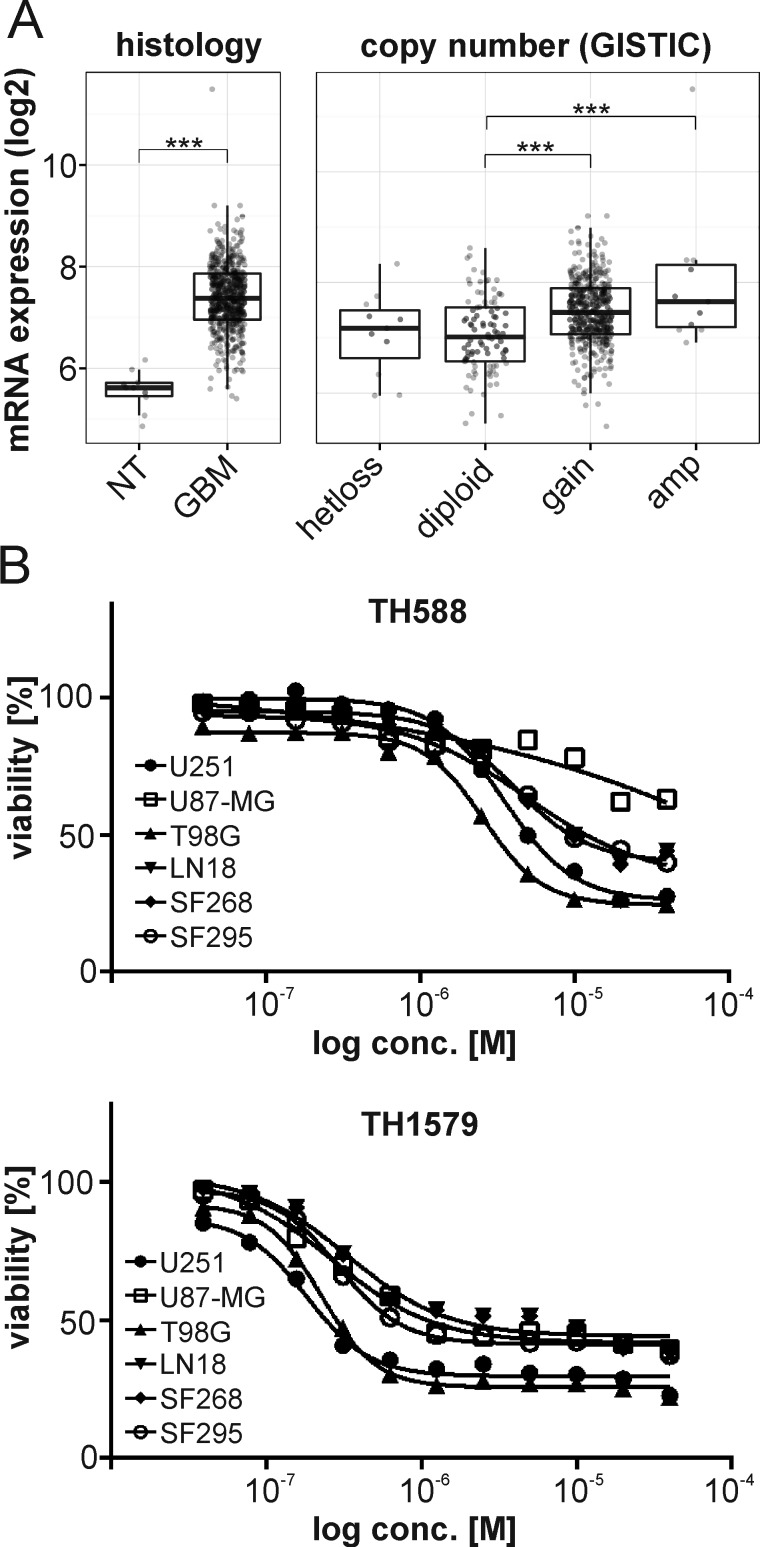
MTH1 is overexpressed in GBM MTH1 mRNA is upregulated in GBM (*n* = 528) compared to non-tumor (NT) samples (*n* = 10), and high levels of MTH1 correlate with its gene copy number in GBM (TCGA dataset). Tukey’s Honest Significant Difference: ***p < 0.001 **A.** Inhibition of MTH1 by the small molecule inhibitors TH588 and TH1579 decreased viability of glioblastoma cell lines **B.**

In order to determine if targeting MTH1 could be a novel strategy to treat GBM, we analyzed available cancer datasets for a potential connection of MTH1 and brain cancer. The TCGA [18, 19], the REMBRANDT [20] and Gravendeel [21] data collections all provided consistent evidence that MTH1 mRNA expression was higher in GBM compared to non-tumor brain tissue and low grade GBM (WHO grade II and III; Figure 1, Supplementary Figure S1). Due to that significant correlation, we investigated the effect of our previously characterized MTH1 inhibitor TH588 [10] and its pharmacologically improved version TH1579 [12] on the survival of six different GBM cell lines. The model compound TH588 decreased potently the viability of five GBM lines after three days of treatment (IC50 < 5.5 µM), only U87-MG required a 5-day treatment for efficient targeting (see Figure 1, Supplementary Figure S2). Interestingly, the levels of MTH1 were lowest in that GBM cell line (see Supplementary Figure S2). Our improved MTH1 inhibitor TH1579, however, significantly decreased viability of all GBM lines after a 3-day treatment with an IC50 < 0.4 µM (Figure 1, Supplementary Figure S2).

### MTH1 requirement is irrespective of GBM aggressiveness

**Figure 2:**
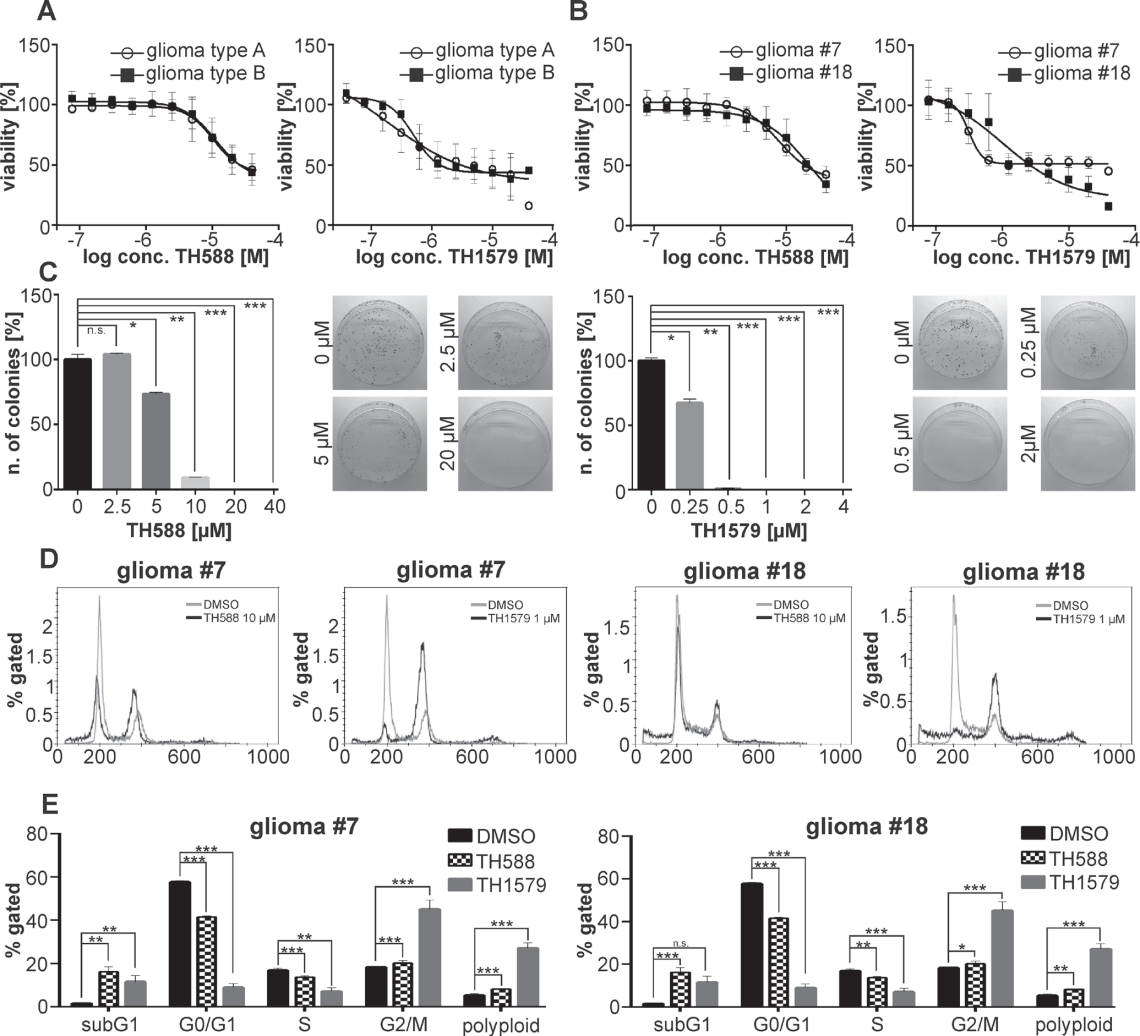
MTH1 inhibitors TH588 and TH1579 target GMB cells independent of aggressiveness The MTH1 inhibitors TH588 and TH1579 target seven patient-derived GBM cultures independent of aggressiveness (type B being more aggressive than type A) **A.** Dose-response curve for TH588 and TH1579 in the most aggressive (#18) and least aggressive (#7) GBM culture **B.** Clonogenic survival of GBM#18 exposed to TH588 and TH1579 **C.** Effect of TH588 and TH1579 on the cell cycle of GBM culture #7 and #18 after 24 hours of exposure **D.**, **E.**

Based on these initial findings, we continued assessing the requirement of functional MTH1 for GBM cell viability in a panel of seven previously characterized GBM lines that can be subdivided in type A and type B, depending on their tumorigenic activity and ability to form spheres *in vitro* [22, 23]. We exposed these patient-derived GBM lines to our MTH1 inhibitors TH588 and TH1579 and observed that the inhibition of MTH1 by either compound significantly decreased viability of all GBM cultures, the improved MTH1 inhibitor TH1579 being more potent compared to the parental compound TH588 (Figure 2A, 2B, Supplementary Figure S3). Calculation of the individual IC50 values revealed that both MTH1 inhibitors killed GBM efficiently independent of their intrinsic aggressiveness (Supplementary Figure S3). Based on the previous characterization of the GBM cultures [22, 23] and their expression level of the GSC surface marker CD133 (Supplementary Figure S6), we chose two GBM cell cultures with either high, type A: culture #18, or low capacity to form neurospheres, type B: culture #7, respectively, for a deeper analysis. We confirmed the requirement of MTH1 for GBM growth and survival by siRNA knock-down (26.5 ± 6.0 % viability compared to control in culture #7, *p* < 0.0001_;_ and 23.7 ± 5.7 % viability compared to control in culture #18, p<0.0001), and further reassured the observed phenotype by using additional three siRNA sequences (see Supplementary Table T3 and Supplementary Figure S4). Moreover, performing clonogenic assays with GBM culture #18 showed that not only inhibition of MTH1 using TH588 and TH1579 significantly reduced the number of colonies, but also that knock-down of MTH1 by siRNA #1 lead to fewer and smaller colonies (Figure 2C, Supplementary Figure S4). We determined additionally the target engagement of MTH1 by TH1579 in intact GBM#18 cells and found it to correspond well to the reduction of viability (Supplementary Figure S4).

**Figure 3:**
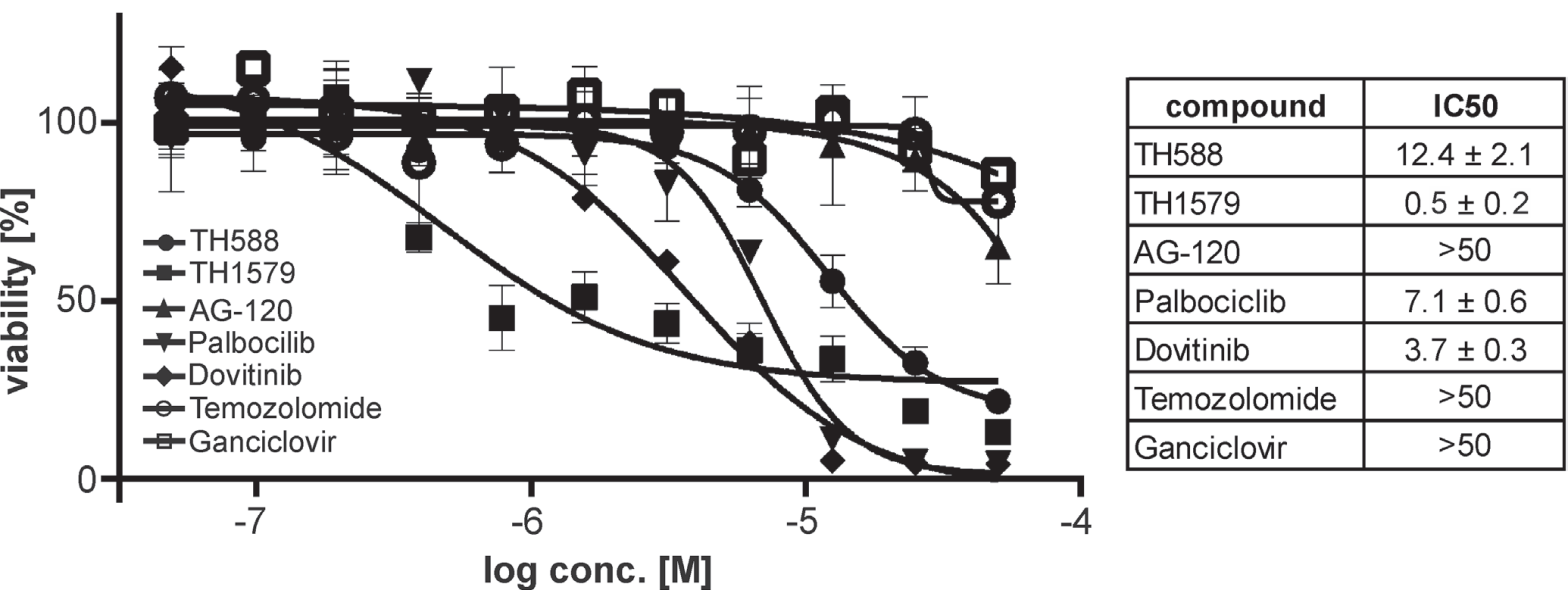
Inhibition of MTH1 is more potent in GBM than standard treatment Dose-response curves for the MTH1 inhibitors TH588 and TH1579 as well as Temozolomide, AG-120, Palbocilib, Dovitinib and Ganciclovir in the GBM line #18.

We continued investigating the effects of TH588 and TH1579 on the cell cycle of GBM cultures 7 and #18. Exposing these GBM cultures to the MTH1 inhibitors for 24 hours depleted both the S and G0/G1 population whereas the fraction of G2/M and polyploid cells increased, most prominently in culture 7 and with the more potent MTH1 inhibitor TH1579 (Figure 2D, 2E). Next, we investigated if MTH1 inhibitors are more potent than today’s standard treatment Temozolomide and Palbociclib, AG-120, Ganciclovir, and Dovitinib which are currently in clinical trial. Indeed, our improved MTH1 inhibitor TH1579 was most potent compared to all other monotherapies in GBM culture #18 (Figure 3)

### Incorporation of oxidized dNTPs into DNA after MTH1 inhibition

**Figure 4:**
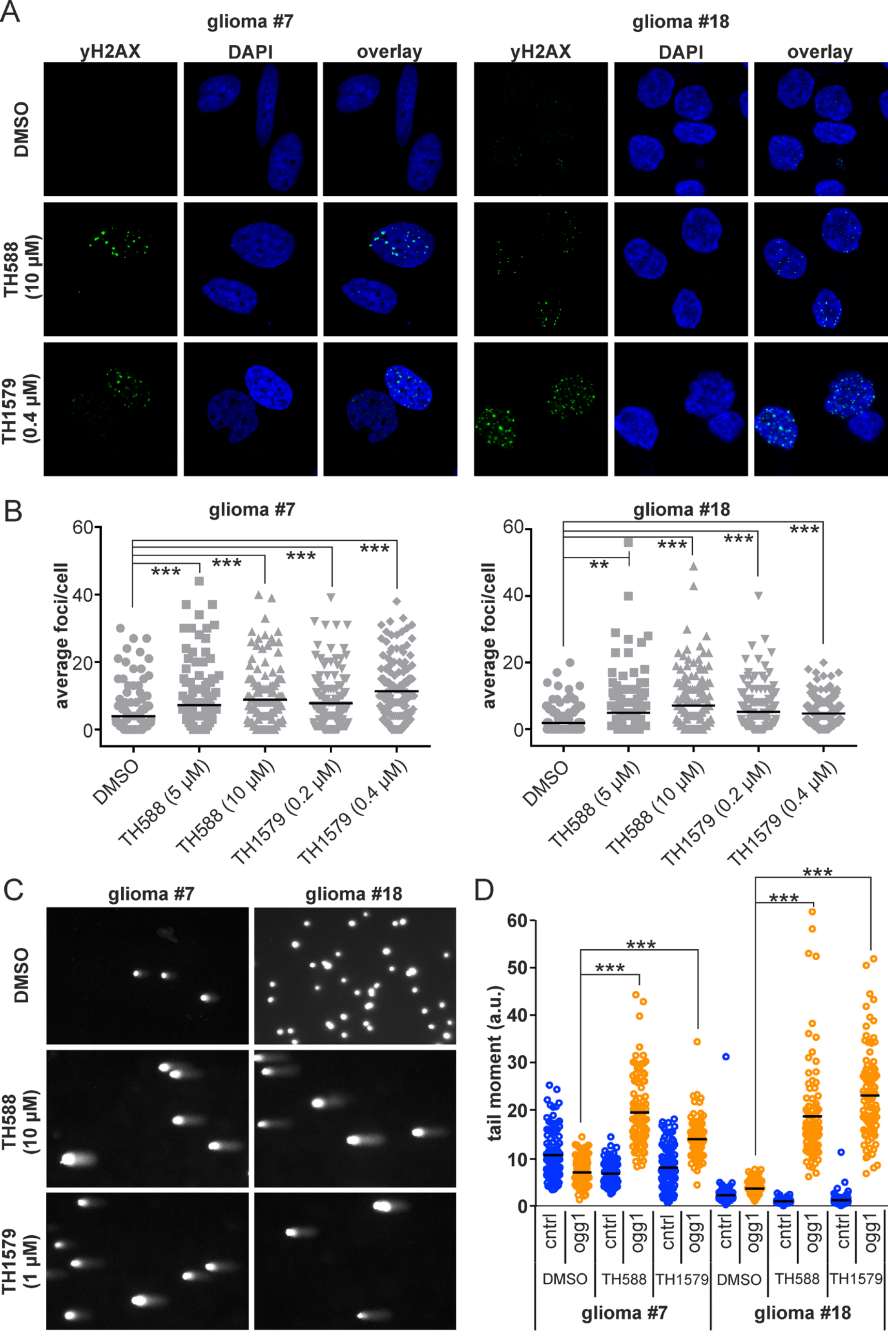
Inhibition of MTH1 induces DNA damage in GBM cells Inhibition of MTH1 by TH588 and TH1579 leads to an increase of yH2AX foci in GBM line #7 and #18 cells after 24 hours **A.** Quantification of yH2AX foci **B.** Comet assay reveals incorporation of 8-oxo-dGTP into DNA of GBM #7 and #18 upon treatment with MTH1 inhibitors for 24 hours **C.** Quantification of 8-oxo-dGTP incorporation, significance calculated with 1-way ANOVA test **D.**

We have previously shown that the loss of functional MTH1 leads to incorporation of oxidized nucleotides, induces DNA damage and subsequently causes cancer cell death [10]. Here, we describe the same phenomenon in GBM culture #7 and #18. As determined by comet assay (Figure 4C, 4D), the inhibition of MTH1 causes incorporation of 8-oxo-dGTP into the DNA of both GBM culture 24 hours after exposure. Moreover, MTH1 inhibition induces DNA damage in both culture #7 and #18 as exemplified by the average number of yH2AX foci per cell which appear after 24 hours of treatment with MTH1 inhibitors (Figure 4A, 4B and Supplementary Figure S5).

### MTH1 inhibition targets CD133+ GBM stem cells *in vitro* and *in vivo*

**Figure 5:**
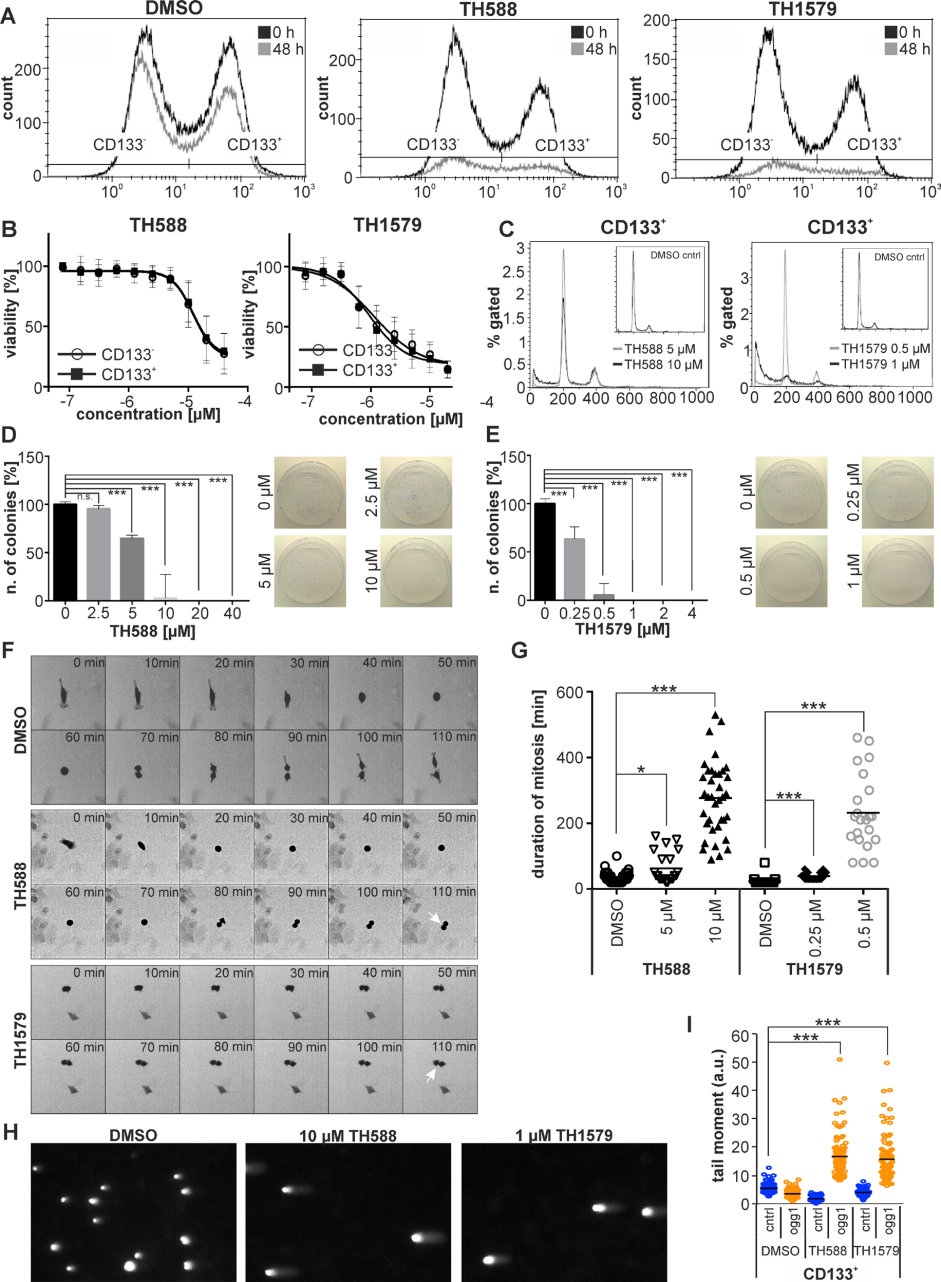
Inhibition of MTH1 targets GBM stem cells Inhibition of MTH1 with TH588 and TH1579 decreases both the CD133^+^ and CD133^-^ cell population of GBM culture #18 equally well **A.** Inhibition of MTH1 by TH588 and TH1579 impairs viability of CD133^+^ and CD133^-^ cells equally well **B.** Exposure to MTH1 inhibitors for 72 hours increases the sub-G1 population of the CD133^+^ cell population of GBM culture #18 **C.** Clonogenic survival of CD133^+^ cells exposed to TH588 **D.** and TH1579 **E.** Mitosis of *SOX2*-GFP^+^ cells is significantly prolonged upon inhibition of MTH1 **F.** Quantification of mitosis duration **G.** Comet assay reveals incorporation of 8-oxo-dGTP into DNA of the CD133^+^ cell population of GBM culture #18 **H.** Quantification of comet assay **I.**

The presence and number of GBM stem cells (GSCs) is one of the major factors which determine aggressiveness and treatment resistance of the tumor bulk, and surface expression of CD133 (PROM1) is regarded as a putative marker for GSCs [24]. In order to elucidate if MTH1 inhibition targets both CD133 positive and negative cells, we subjected culture #18 to both TH588 and TH1579 before analysis using flow cytometry. After 48 hours of treatment we observed that, albeit an overall decreasing cell count, the proportion of CD133^+^ and CD133^-^ cells remained stable during the course of the treatment, which indicated that MTH1 inhibitors target CD133^+^ and CD133^-^ cells equally well (Figure 5A, Supplementary Figure S9B). Importantly, we observed no significant decrease of the CD133^+^ population during the experimental timeframe in untreated cells, hence can exclude spontaneous differentiation of putative GSCs (Supplementary Figure S9D). To provide further evidence that MTH1 inhibitors target GSCs, we enriched a CD133^+^ cell population from culture #18 by magnetic-activated cell sorting (MACS) to about 79 % (Supplementary Figure S9A, B) and exposed it to MTH1 inhibitors. After 72 hours of treatment, we observed that MTH1 inhibitors targeted the fraction of putative GSCs equally well compared to the CD133^-^ cell population (IC50_TH588_ = 12.1 ± 2.9µM and IC50_TH1579_ = 0.93 ± 0.79 µM for the CD133^+^ fraction; IC50_TH588_ = 12.1 ± 2.7 µM and IC50_TH1579_ = 1.1 ± 1.0 µM for the CD133^-^ fraction; see Figure 5B). Besides measuring viability, we also determined the clonogenic survival of the CD133^+^ population upon TH588 and TH1579 treatment, and found it to be severely impaired by the loss of functional MTH1 (Figure 5D, 5E). FACS analysis verified that the CD133^+^ population did not differentiate during the course of the experiment (Supplementary Figure S4B). The expression of the transcription factor *SOX2* has been associated with GBM aggressiveness and GSCs [23, 25]. Based on a previously characterized construct [26], we stably transfected GBM culture #18, which expresses GFP under the *SOX2* promoter. Live cell imaging of this GBM culture revealed a significantly prolonged mitosis of *SOX2*-GFP^+^ cells upon exposure to TH588 and TH1579 (Figure 5F, 5G), adding further evidence that MTH1 inhibition targets GSCs. Furthermore, we have analyzed the effect of TH588 and TH1579 on the cell cycle of the CD133^+^ population of GBM #18 and found the sub-G1 population to significantly increase after exposing to MTH1 inhibitors for 72 hours (Figure 5C, Supplementary Figure S10). Lastly, we were able to reveal that incorporation of 8-oxo-dGTP into the CD133^+^ population of GBM #18 significantly increases upon MTH1 inhibition (Figure 5H, 5I).

**Figure 6:**
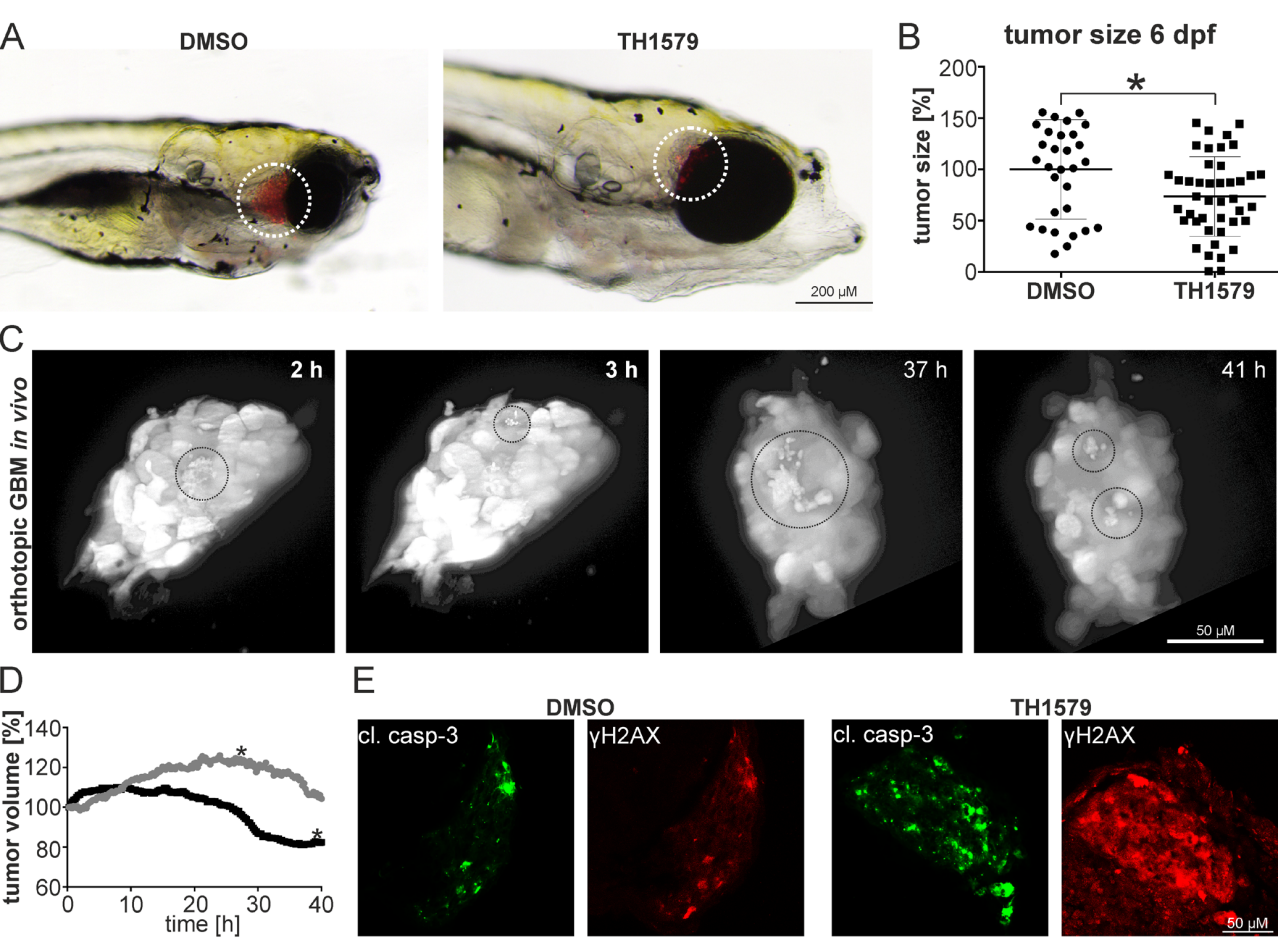
MTH1 inhibitors target GBM and GBM stem cells *in vivo* GBM #18-CMV-LUC cell enriched for the CD133^+^ population have been orthotopically injected into zebrafish embryos. 6 days post injection, embryos exposed to 50 µM TH1579 displayed smaller tumors **A.** Quantification by luminescence measurements in single embryos showed 26.4 % smaller tumors in TH1579 treated embryos (*n* = 43) compared to DMSO controls (*n* = 31; *p* = 0.011) **B.** Still images of real-time light sheet microscopy on orthotopic xenotransplants exposed to 50 µM TH1579 apoptotic cells encircled **C.** Determination of tumor volume of xenotransplant. Grey circle: DMSO control, black circle TH1579 treated (tumor shown in (C). The asterisks mark the time-point when the transplants started leaving the focal plane **D.** Immunocytochemistry on cleaved caspase as well as y-H2AX in orthotopic xenotransplants treated for 5 days with 50 µM TH1579 or DMSO **E.**

The zebrafish is a powerful pre-clinical animal model and its relevance for glioblastoma research is well documented [27, 28]. We generated luciferase expressing GBM#18 cells (#18-CMV-LUC), enriched the CD133^+^ putative stem cell population to >85 % (Supplementary Figure S11) and transplanted those orthotopically into the brain of zebrafish embryos. After 5 days of treatment, we determined the tumor size by luminescence measurements in single embryos and found that treatment with 50 µM TH1579 (*n* = 43) resulted in 26.4 % smaller tumors compared to DMSO controls (*n* = 31; *p* = 0.011; Figure 6A, 6B). One prominent advantage of the zebrafish model is its transparency which allows assessing tumor growth and the effect of anti-cancer compounds *in vivo* and real time. We therefore transplanted the GBM line U343-MGA:GFP orthotopically into zebrafish embryos, exposed to 50 µM TH1579 and followed the tumor for 48 hours intracranially using light-sheet microscopy. In this *in vivo* setting we were not only able to detect numerous cells undergoing cell death, but also that the overall tumor volume decreased by ∼25 % within 35 hours of treatment while the non-treated tumor increased its volume by ∼ 20 % (Figure 6C, movie S1). Moreover, we confirmed our data in cell culture as we could verify that exposure to 50 µM TH1579 lead to an increased expression of cleaved caspase 3 as well as the DNA damage marker y-H2AX in orthotopically transplanted tumors compared to DMSO controls (Figure 6E).

## DISCUSSION

Current therapeutic options for neurological cancers including GBM are very limited and lethal tumor recurrence is unavoidable. It has been suggested that amplification of chromosome 7 together with loss of chr10 are very early genetic events GBM ontogeny, potentially because of the tumor driver *PDGFA* on chromosome 7 [29, 30]. Interestingly, the *MTH1-*gene is also localized on chr7 (7p22) and indeed, by analyzing three different datasets, we found *MTH1* transcripts to be significantly overexpressed in GBM as well as its expression levels to be correlated to GBM aggressiveness and proliferative potential, a finding that was recently described [31]. Besides, there is also direct immunohistological evidence that MTH1 protein levels are elevated in human brain tumors with highest levels found in aggressive GBM [13]. Based on these observations, we thought to investigate if GBM growth and survival depends on the presence of functional MTH1 protein.

We performed our initial investigations in a collection of seven patient-derived GBM cultures that have been previously characterized [22, 23]. Applying four different siRNAs as well as two different in-house developed MTH1 inhibitors, we observed that suppression as well as inhibition of MTH1 efficiently killed all analyzed patient-derived GBM cultures, irrespectively of their aggressiveness. Furthermore, we found the target engagement of TH1579 for MTH1 in intact GBM#18 cells closely corresponding to the effect on cell viability, indicating specificity of the compound. During preparation of this manuscript two other groups independently confirmed our findings that MTH1 is indispensable for GBM growth and survival [31, 32]. Spurred by these initial results, we continued with an in-depth analysis of our most aggressive GBM cell culture #18 and the least aggressive representative culture #7.

Cancer cells and GBM cells in particular suffer from a high redox pressure and are therefore dependent on specific detoxification enzymes including MTH1, which degrades oxidized nucleotides including 8-oxo-dGTP. Indeed, after we inhibited MTH1 with either TH588 or TH1579, we were able to detect specific 8-oxo-guanine lesions in the DNA of the analyzed GBM cultures using a modified comet assay. As anticipated, we also observed an increase of general DNA damage following exposure to MTH1 inhibitors, most likely due to the incorporation of 8-oxo-dGTP, which is known to eventually lead to cellular senescence through persistent DDR signaling [33]. Since it has recently been published that DNA synthesis can occur at common fragile sites (CFSs) during mitosis of cells suffering from replicative stress, *i.e*. cancer cells [34], it will be interesting to investigate if 8-oxo-dGTP might be incorporated into these fragile sites in absence of functional MTH1 with fatal consequences. Besides its DNA damaging potential, it has been published that modified guanine nucleotides including modifications on the 8^th^ position may affect the polymerization of tubulin *in vitro* and *in vivo* [35, 36]. Since we have observed that our MTH1 inhibitors induce a G2/M arrest as well as lead to polyploid cells, we are currently exploring if these effects are based on the 8-oxo-dGTP interference with tubulin dynamics. The vast majority of GBM reappears after initial treatment, most often due to overexpression of O6-methyl-guanidine methyltransferase (MGMT) which confers resistance to Temozolomide [1]. Importantly, despite expressing very high levels of MGMT (see Supplementary Figure S7) and being resistant to Temozolomide, we show that MTH1 inhibitors efficiently target GBM cultures #7 and #18. Since MTH1 inhibitors potently decreased viability of these resistant cultures, it is tempting to speculate that MTH1 inhibition could be used as novel strategy to target recurring, Temozolomide resistant GBMs.

Although the cancer stem cell hypothesis remains controversial, there is a large body of evidence supporting a hierarchical architecture of GBM with GSCs at the apex [37, 38]. GSCs drive aggressiveness of the tumor, confer resistance to current treatments and are the main cause for disease recurrence [1]. Since we found that our MTH1 inhibitors target the two different GBM cultures potently and independent of their aggressiveness, we investigated if GSCs are also dependent on functional MTH1. The surface protein CD133 is currently one of the best available markers for putative GSCs [24], thus we fractionized culture #18 with MACS accordingly and present several lines of evidence that MTH1 inhibitors decrease survival of the CD133 enriched and depleted fraction of GBM culture #18 equally well. Moreover, our inhibitors induced a dramatic prolongation of mitosis as well as lead to incorporation of 8-oxo-dGTP into the DNA of culture #18 cells expressing *SOX2*, a stemness marker also upregulated in GSCs [23, 25]. We transplanted the CD133^+^ enriched fraction intracranially into zebrafish embryos, which is a clinically relevant and ethically sound model for GBM [27, 28]. By that, we were not only able to confirm the ability of MTH1 inhibitors to target GSCs *in vivo*, but also to monitor *in vivo* and in real time how GSC cells died due to this anti-cancer drug*.* Although the transplanted zebrafish embryos were freely swimming in a 50 µM solution of TH1579, the actual drug exposure of the tumor was likely significantly lower due to limited uptake of this small molecule into the zebrafish. However, we recently confirmed good pharmacokinetic parameters including good oral availability in mouse [12]. As the cellular fraction depleted for CD133 did not form stable intracranial tumors, likely due to the absence of stem cells, we followed additionally a non-orthotopic implantation approach to show that inhibition of MTH1 targets both GBM as well as GSCs (see Supplementary Figure S12). It is well established that GSCs preferentially localize in the perinecrotic hypoxic area [39, 40], which contributes to regulate their tumorigenic capacity [41]. Hypoxia in general and the perinecrotic region specifically are associated with oxidative stress [42-44], and GSCs were indeed described to suffer from higher oxidative pressure compared to non-GSCs [45]. We have recently found that hypoxia and the tumor redox environment determine sensitivity to MTH1 inhibition [16], therefore the elevated redox pressure present in GSCs might explain their addiction to functional MTH1.

In summary, we have presented evidence that inhibition of MTH1 might represent an efficient strategy to target GBM.

## MATERIALS AND METHODS

### Data analysis

GBM expression data were analyzed through the GlioVis data portal (http://gliovis.bioinfo.cnio.es) (M. Squatrito, manuscript in preparation). Multiple comparisons were performed by ANOVA test followed by Tukey’s Honest Significant Difference post-hoc analysis.

### Antibodies and chemicals

The following antibodies were used: mouse anti-yH2AX (Millipore), mouse anti beta-actin (Abcam) and IRDye 680RD donkey anti-mouse (LI-COR), rabbit anti-cleaved caspase 3 (Abcam), mouse anti y-H2AX (Millipore), anti-rabbit/555 (Invitrogen) and anti-mouse/488 (Life technologies). Chemicals were purchased from Sigma-Aldrich. MTH1 inhibitors were synthesized in-house and dissolved in DMSO to 10 mM [10, 12].

### Cell culture, comet and viability assay

All cell lines used have been obtained from ATCC during the last four years; regular mycoplasma tests have been performed. Generation, authentication and propagation of patient-derived GBM cell cultures have been described elsewhere [23]. Cell viability measurements using the MTT or resazurin assay were performed as described [10]. The modified comet assay has been described in detail before [10]. Briefly, the 8oxodGTP specific exonuclease Ogg1 has been used to induce DNA strand breaks at 8-oxo-dGTP positions which could then specifically be detected using the comet assay. The *SOX*2-GFP reporter cells have been established with a lentivirus based system following standard protocols and using a previously described construct [26]. Lipofectamine was used for siRNA knock-downs. SiRNA sequences see table S2 and Supplementary Figure S7. For clonogenic assays using MTH1 inhibitors, 500 cells were seeded in 10 cm dishes and treated as specified. After 10 days, colonies were stained with methylene blue and counted manually. Neither medium nor treatment was exchanged during the course of the experiment. For clonogenic assays using siRNA, cells were treated with siRNA for 24 hours before seeding, then propagated and stained as above. Colony number and size was determined using the cell profiler software.

### CETSA- Alpha-screen

Glioma#18 cells were grown in 6-well plates and treated with TH1579 at different concentrations for 1 hour at 37 °C. After trypsinization cells were collected by centrifugation and resuspended in 50 µl of TBS, pH 8.0, and protease inhibitor COMPLETE (Roche). Cell suspensions were transferred to a 96-well PCR plate (Agilent) and heated to 58 °C followed by addition of lysis buffer (SureFire Ultra5x, Perkin Elmer). After 20 minutes lysis at room temperature lysates were transferred to an assay plate (Proxiplate, Perkin Elmer) and the amounts of stabilized MTH1 were analyzed using Alpha Lisa immunoassay[46]. Cell lysates were incubated with two MTH1 antibodies diluted in immuno assay buffer (Perkin Elmer), mouse anti-MTH1 (Ls-c197715, LS-Bio), 1:20000 and rabbit anti-MTH1 (polyclonal, in house produced), 1:2000, for two hours at room temperature. Anti-mouse IgG alpha donor beads 10 µg/ml and anti-rabbit IgG acceptor beads, 10 µg/ml (Perkin Elmer) were added to the assay plate followed by incubation at room temperature overnight. Plates were read using Envision plate reader (Perkin Elmer), the light emission was directly proportional to the amount of stabilized MTH1

### Magnetic-activated cell sorting

Cells were harvested with Accutase (Sigma) and resuspended in PBS containing 0.5 % BSA and 2 mM EDTA. Sorting was performed using the CD133 MicroBead Kit , efficiency of cell sorting was analyzed by flow cytometry using the Labeling Check Reagent-PE (all Miltenyi Biotec).

### Flow cytometry

Harvested cells were resuspended in PBS containing 2 % FBS and labelled with anti-CD133/1-APC (Miltenyi Biotec) in presence of human Fc-Block (BD Biosciences). The mouse IgG1 isotype control antibody conjugated to APC (Milteny Biotec) served as control (see Supplementary Figure S6). For cell cycle analysis, cells were pulsed for 4 h (culture #7) or 0.5 h (culture #18) with 10 µM EdU followed by fixation and incubation with mouse anti-yH2AX overnight. EdU was detected based on a Click-iT EdU system, DNA was stained using Hoechst 33342 according to standard protocols.

### RNA isolation, reverse transcription and quantitative real-time PCR

RNA was isolated and cDNA was synthesized with the Maxima First strand cDNA Synthesis Kit and RTqPCR was run using the Luminaris Color HiGreen qPCR Master Mix (all ThermoScientific) on a Rotorgene (Qiagen). Relative gene expression levels normalized to beta actin were calculated according to the 2^-ΔΔCT^ method. Primer were purchased pre-verified (KICqStart, Sigma) and always span an exon-intron junction (see table S1).

### Zebrafish maintenance, injections and exposure

Zebrafish (TL) were raised and staged according to standard protocols. Cells transplanted into zebrafish were transferred to stem cell medium (Neurobasal medium/DMEM/F12 mixture containing B27 and N2 supplements as well as 10 ng/ml bFGF and 20 ng/ml EFG) 7 days before injection. Labelling and transplantation has been described before [47]. 24 hours after injection, treatment was started. All experiments were performed in accordance to the international and local ethical guidelines (N207/14).

### Generation of luciferase expressing cells and luciferase measurement

The glioblastoma line #18 was stably transfected with pLenti CMV Puro LUC (Addgene) using routine protocols. To measure luminescence, 1 embryo/ well was plated in 96 well plates and lysed (10 % glycerol, 1 % Triton-X100, 8 mM MgCl2, 1 mM DTT, 25 mM Tris-phosphate pH7.8) for 20 min followed by addition of luciferase detection buffer (1 mM DTT; 1 mM ATP, 0.3 mg/ml luciferin potassium salt, 25 mM Tris-phosphate pH 7.8) and light detection.

### Live cell imaging

The *SOX*2-GFP reporter cells were seeded in 96 well black/clear plates at a concentration of 6000 cells per well and subjected to treatment the day after. Within 30 min after addition of MTH1 inhibitors, live cell imaging was performed using the Image Express microscope (Molecular Devices) at 37° C in 5 % CO_2_ atmosphere. Images were acquired with a 20x lens every 10 min for 24 hours. The images of each channel were assembled into AVI-movies using the MetaXpress software and the cell division was analyzed with ImageJ

### Light sheet microscopy

24 hpf embryos were embedded in 1% low melt agarose in a glass capillary and extruded into a sample chamber containing E3 medium supplemented with MS222. Images were acquired using a Light Sheet Z.1 (Carl Zeiss, Germany) with a water dipping 20X detection objective (W-Plan-APOCHROMAT-1.0NA) and dual side 10X illumination objectives (LSFM, 0.2NA). Samples were illuminated from a single side and Z-stacks were acquired every 15 min using a 1.2X optical zoom, 4.13 µm light sheet thickness and 2 µm Z-interval. Tumor volume was measured using the Countour Surface tool in Imaris 8.3.1 (Bitplane). For visualization, max intensity projections were produced and drift was corrected using a rigid body transformation in the ImageJ plugin, StackReg [48]

### Immunocytochemistry

Cells were seeded in 6 well plates and subjected to treatment 24 hours after. After exposure, cells were treated for immunocytochemistry according to standard protocols. Embryos were prepared for cryo-sections as described previously [49] and sections were stained using routine protocols.

### Documentation and statistical analysis

Pictures were taken with a Leica LSM 780 confocal microscope or a Leica MZ16 microscope equipped with a Leica DCF3000FX camera. Image data was processed with GIMP or ImageJ without obstructing any original data. Experiments have been performed at least in triplicates, the results are expressed as mean ± SD. Statistical significance was determined using the two-tailed student’s t-test unless otherwise stated with the following *p* values considered significant: *: *p* <0.05; **: *p* <0.001; ***: *p* < 0.0001.

## SUPPLEMENTARY MATERIALS FIGURES, TABLES AND VIDEO




