# Correction: Glioblastoma and glioblastoma stem cells are dependent on functional MTH1

**DOI:** 10.18632/oncotarget.27484

**Published:** 2020-02-25

**Authors:** Linda Pudelko, Pegah Rouhi, Kumar Sanjiv, Helge Gad, Christina Kalderén, Andreas Höglund, Massimo Squatrito, Alberto J. Schuhmacher, Steven Edwards, Daniel Hägerstrand, Ulrika Warpman Berglund, Thomas Helleday, Lars Bräutigam

**Affiliations:** ^1^ Department of Medical Biochemistry and Biophysics, Science for Life Laboratory, Division of Translational Medicine and Chemical Biology, Karolinska Institutet, Stockholm, Sweden; ^2^ Cancer Cell Biology Programme, Seve Ballesteros Foundation Brain Tumor Group, Centro Nacional de Investigaciones Oncológicas, CNIO, Madrid, Spain; ^3^ Department of Applied Physics, Science for Life Laboratory, Royal Institute of Technology, Stockholm, Sweden; ^4^ Department of Oncology-Pathology, Karolinska Institutet, Stockholm, Sweden; ^5^ Department of Oncology, Lab of Tumor Inflammation and Angiogenesis, KU Leuven, Leuven, Belgium

**This article has been corrected:** The Funding information has been updated. The complete Funding list is shown below:

**FUNDING**

This work was supported by the Karolinska Institutes KID funding (LP), the Seve Ballesteros Foundation to MS, the Marie Curie foundation (CIG-618751 MS), the Knut and Alice Wallenberg Foundation (KAW2014.273 TH), the Swedish Foundation for Strategic Research (RB13-0224 TH), the Swedish Cancer Society (TH), the Swedish Research Council (2015-00162 TH), the Göran Gustafsson Foundation (TH), the Swedish Children’s Cancer Foundation (to TH), the Swedish Pain Relief Foundation (PR20140048 TH), the Torsten and Ragnar Söderberg Foundation (TH), and the European Research Council (TAROX-695376).

Original article: Oncotarget. 2017; 8:84671–84684. 84671-84684. https://doi.org/10.18632/oncotarget.19404

