# Supplementary material for: Glioblastoma and glioblastoma stem cells are dependent on functional MTH1
Source: Oncotarget. 2017 Jul 20;8(49):84671–84. doi: 10.18632/oncotarget.19404 (PMC5689565; doi:10.18632/oncotarget.19404)
Supplement: Supplementary file 1 [file oncotarget-08-84671-s001.pdf]

Glioblastoma and glioblastoma stem cells are dependent on functional MTH1

SUPPLEMENTARY MATERIALS

Supplementary Table S1: RTqPCR primer sequences

Target	Sequence 5’-3’
**beta actin, forward**	CCTGGCACCCAGCACAAT
**beta actin, reverse**	GGGCCGGACTCGTCATACT
**MGMT forward**	CACGAAATAAAGCTCCTGG
**MGMT reverse**	GACTCTTGCTGGAAAACG

Supplementary Table S2: siRNA sequences

Target	Name	Sequence 5' - 3'
**MTH1**	siRNA #1	CTCCTGCTTCAGAAGAAGAAA
	siRNA #2	CCGGGTTTCATCTGGAATTAA
	siRNA #3	CGAGTTCTCCTGGGCATGAAA
	siRNA #4	TCAGGACACCATCCTGGACTA

Supplementary Table S3: viability after siRNA treatment

		viability compared to cntrl siRNA in %	ttest
**glioma #7**	siRNA #1	26.5 ± 6.0	1.6E-13
	siRNA #2	43.7 ± 9.6	2.8E-10
	siRNA #3	29.3 ± 6.2	3.2E-13
	siRNA #4	26.3 ± 7.6	5.2E-13
**glioma #18**	siRNA #1	23.7 ± 5.7	1.3E-07
	siRNA #2	47.3 ± 12.7	3.6E-06
	siRNA #3	57.5 ± 9.6	0.00003
	siRNA #4	54.9 ± 16.8	2.7E-06

Supplementary figures

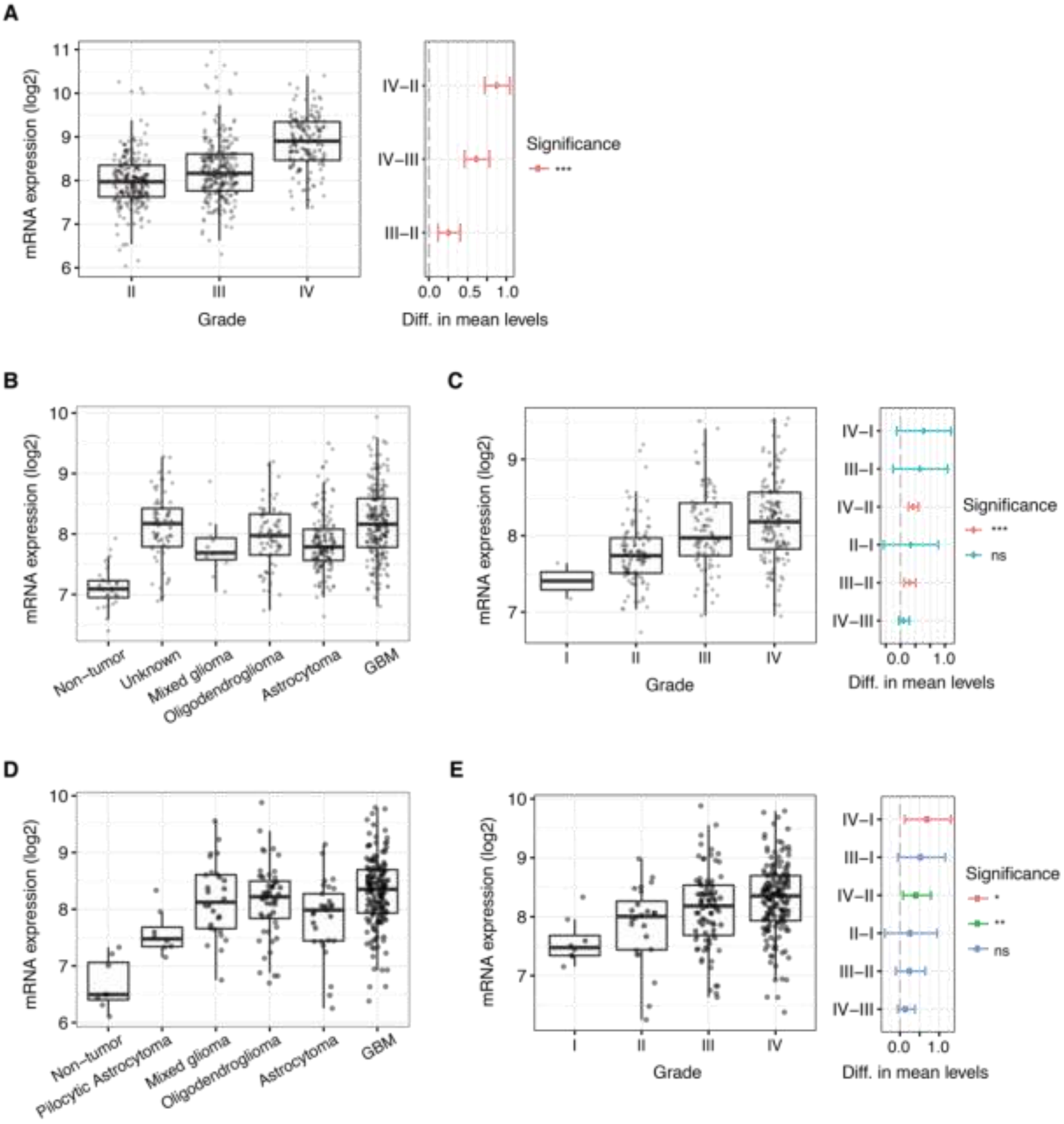

Supplementary Figure 1: MTH1 is upregulated in glioblastoma

MTH1 RNA-seq expression data of the TCGA pan-glioma dataset (A). MTH1 microarray expression data of the REMBRANDT (B-C) and Gravendeel (D-E) datasets, separated by histology or grade. (A, C, and E, right panels). Tukey's Honest Significant Difference: ***p < 0.001; **p < 0.01; *p < 0.05; ns, not significant.

A

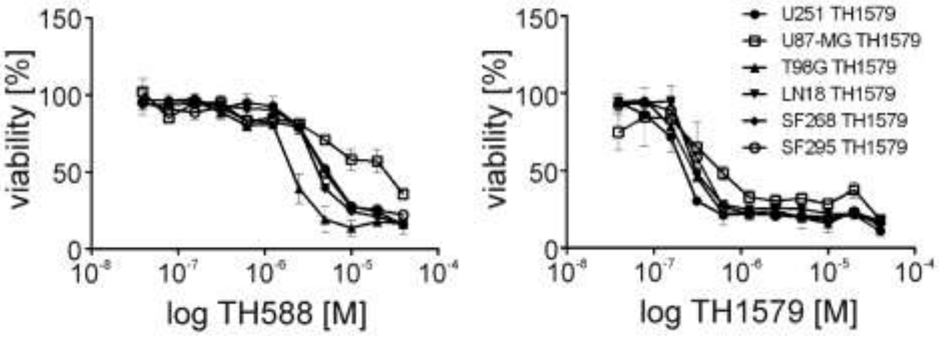

B

	IC50 in µM			
	72 hours treatment		120 hours treatment	
	TH588	TH1579	TH588	TH1579
**U251**	3.49 ± 1.07	0.14 ± 1.10	4.47 ± 1.05	0.19 ± 1.04
**U87-MG**	380.1 ± n.a.	0.28 ± 1.13	6.12 ± 4.51	0.41 ± 1.24
**T98G**	2.45 ± 1.08	0.21 ± 1.05	1.92 ± 1.01	0.24 ± 1.06
**LN18**	3.99 ± 1.087	0.38 ± 1.09	3.75 ± 1.03	0.28 ± 1.04
**SF268**	5.14 ± 1.22	0.26 ± 1.10	5.15 ± 1.13	0.29 ± 1.19
**SF295**	5.47 ± 1.3	0.29 ± 1.3	4.21 ± 1.08	0.31 ± 1.03

C

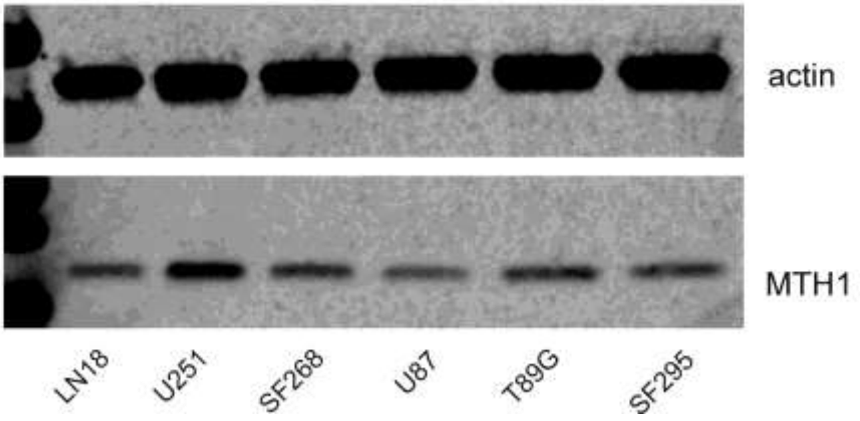

Supplementary Figure 2: MTH1 inhibitors efficiently target glioblastoma cell lines

Six different GBM cell lines were exposed to the MTH1 inhibitors TH588 and TH1579 for 5 days and followed by survival measurement (A). IC50 values for 3 days and 5 days of treatment (B). Western blot analysis of protein levels in the GBM cell lines tested (C).

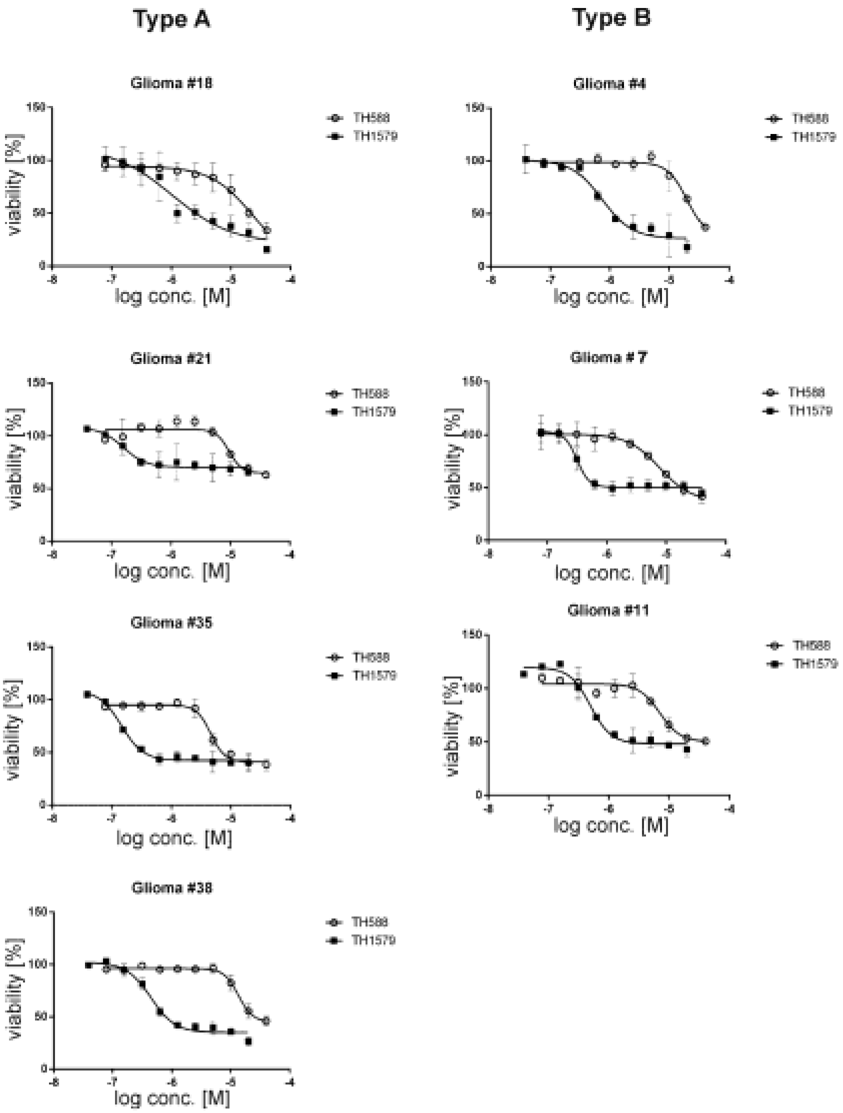

# glioma	Type	IC50 TH588 [µM]		IC50 TH1579 [µM]	
		single	combined	single	combined
4	Type B	19.9	11.6 ± 7.2	0.7	0.5 ± 0.2
7		7.8		0.3	
11		7.1		0.5	
18	Type A	12.5	10.0 ± 3.5	0.65	0.3 ± 0.2
21		9.5		0.2	
35		4.6		0.1	
38		13.5		0.4	

ttest for TH588 glioma type A vs. glioma type B:	0.87
ttest for TH1579 glioma type A vs. glioma type B:	0.78

Supplementary figure 3: MTH1 inhibitors kill glioblastoma lines independent of aggressiveness

Survival curves of patient-derived GBM cell lines exposed to the MTH1 inhibitors TH588 and TH1579 (A). IC50 values for TH588 and TH1579 for the individual GBM lines (B).

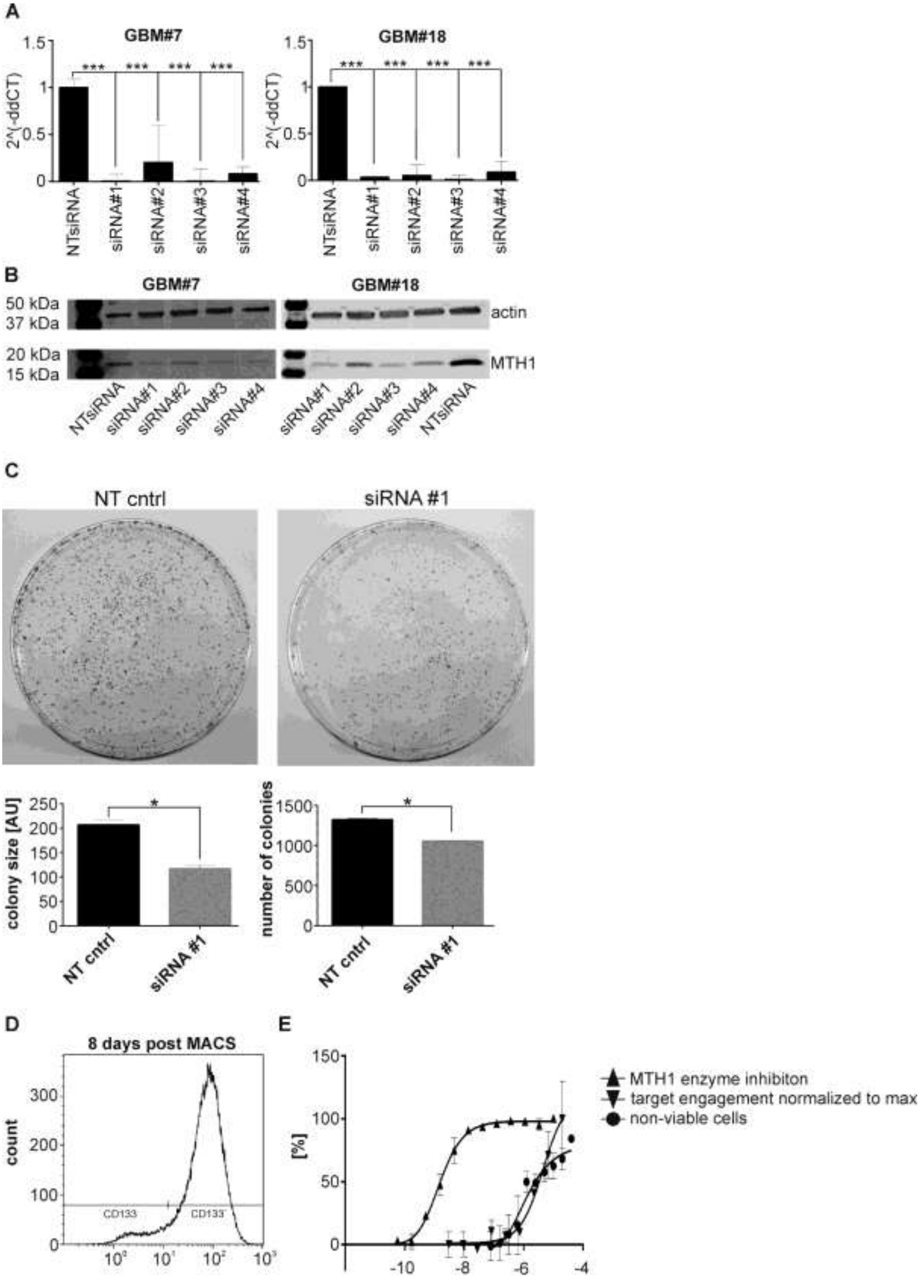

Supplementary Figure 4: Loss of functional MTH1 impairs proliferation of GBM culture #18

MTH1 mRNA levels after siRNA treatment for 3 days (A). MTH1 protein levels after siRNA treatment for 3 days (B). Clonogenic survival of GBM #18 after MTH1 knock-down using siRNA #1 (C). FACS plot showing the proportion of $CD133^+$ cells after termination of the clonogenic survival experiment, i.e. 8 days post MACS sorting (D). Enzymatic inhibition of MTH1 upon TH1579 treatment, effect of TH1579 on viability and MTH1 target engagement of TH1579 in GBM#18 cells following 1 h treatment using CETSA (E).

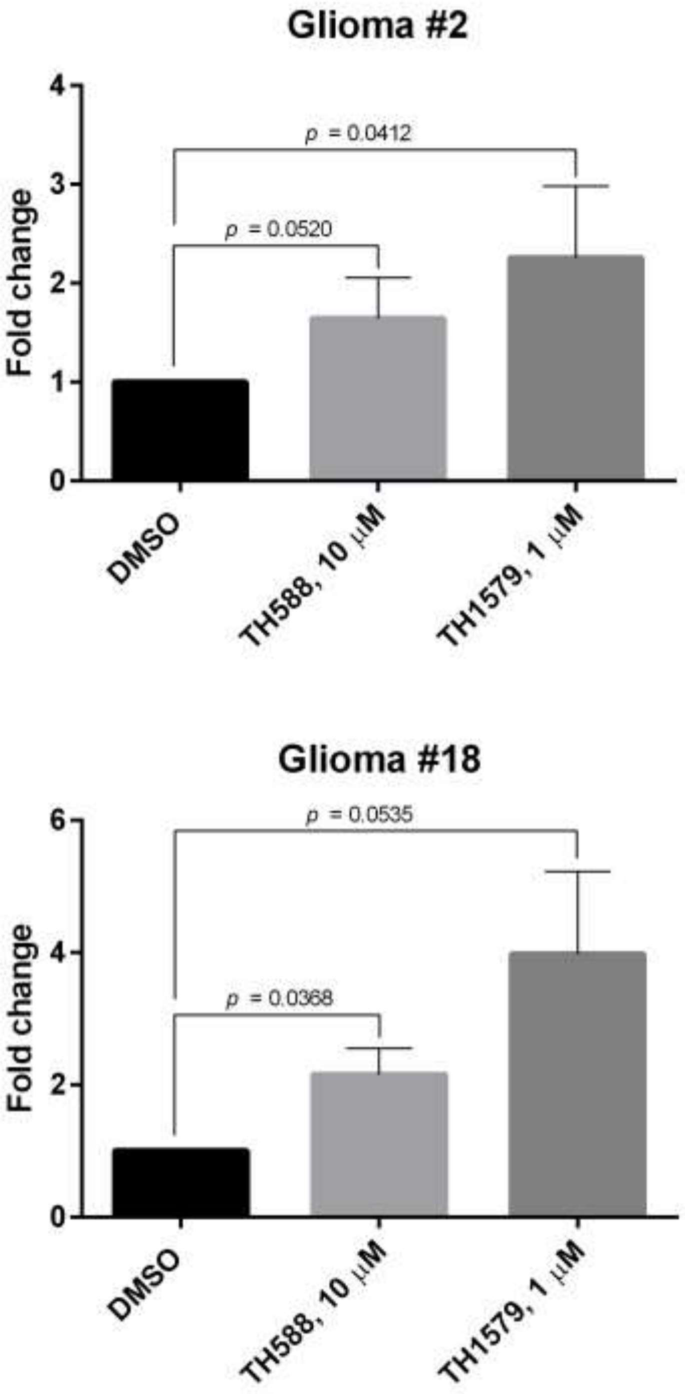

Supplementary Figure 5: **Loss of functional MTH1 induces DNA damage**

FACS analysis of yH2AX staining after MTH1 inhibition.

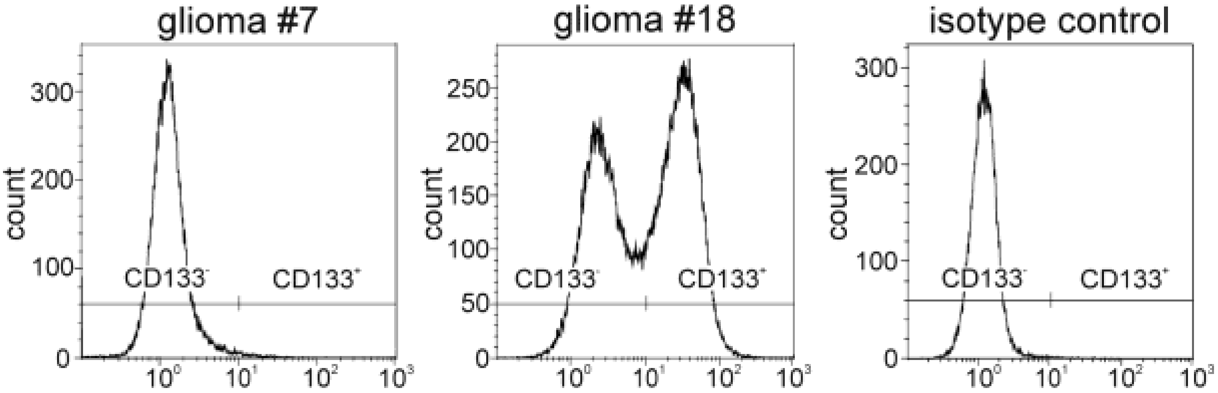

	glioma #7	glioma #18	isotype control
CD133+ fraction in %	0.99	53.30	0.30
CD133- fraction in %	99.01	46.70	99.70

Supplementary Figure 6: Surface expression of CD133 and antibody isotype control

The fraction of cells expressing CD133 on the surface was determined by FACS and compared to the isotype control.

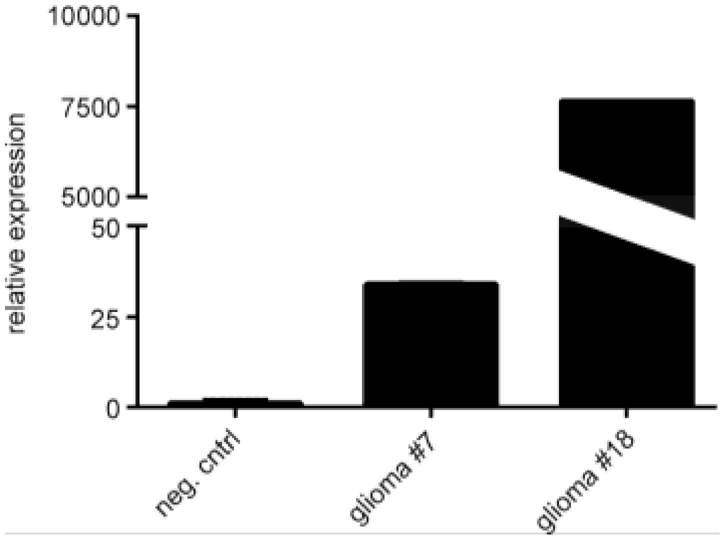

Supplementary Figure 7: MGMT expression in glioblastoma

MGMT expression in GBM #7 and #18. K562 was used as negative control.

```
                                              #3
  1 ggtcagaggc cacgcccccg gaagcggcgg tgcagaaccc agggaccatg ggcgcctcca
 61 ggctctatac cctggtgctg gtcctgcagc ctcagcgagt tctcctgggc atgaaaaagc
121 gaggcttcgg ggccggccgg tggaatggct ttgggggcaa agtgcaagaa ggagagacca
181 tcgaggatgg ggctaggagg gagctgcagg aggagagcgg tctgacagtg gacgccctgc
241 acaaggtggg ccagatcgtg tttgagttcg tgggcgagcc tgagctcatg gacgtgcatg
301 tcttctgcac agacagcatc caggggaccc ccgtggagag cgacgaaatg cgcccatgct
361 ggttccagct ggatcagatc cccttcaagg acatgtggcc cgacgacagc tactggtttc
     #1                                                  #4
421 cactcctgct tcagaagaag aaattccacg ggtacttcaa gttccagggt caggacacca
481 tcctggacta cacactccgc gaggtggaca cggtctagcg ggagcccagg gcagcccctg
541 ggcaggagac gtggctgctg aacagccgca aaccatcttc acctgggggc attgagtggc
         #2
601 gcagagccgg gtttcatctg gaattaactg gatggaaggg aaaataaagc tatctagcgg
661 tgaaaaaaaa aaaaaaaaaa aaaaaaaaaa aa
```

Supplementary Figure 8: siRNA targets in MTH1 coding sequence

The boxed sequences show the targets of the siRNAs used to knock-down MTH1 in GBM

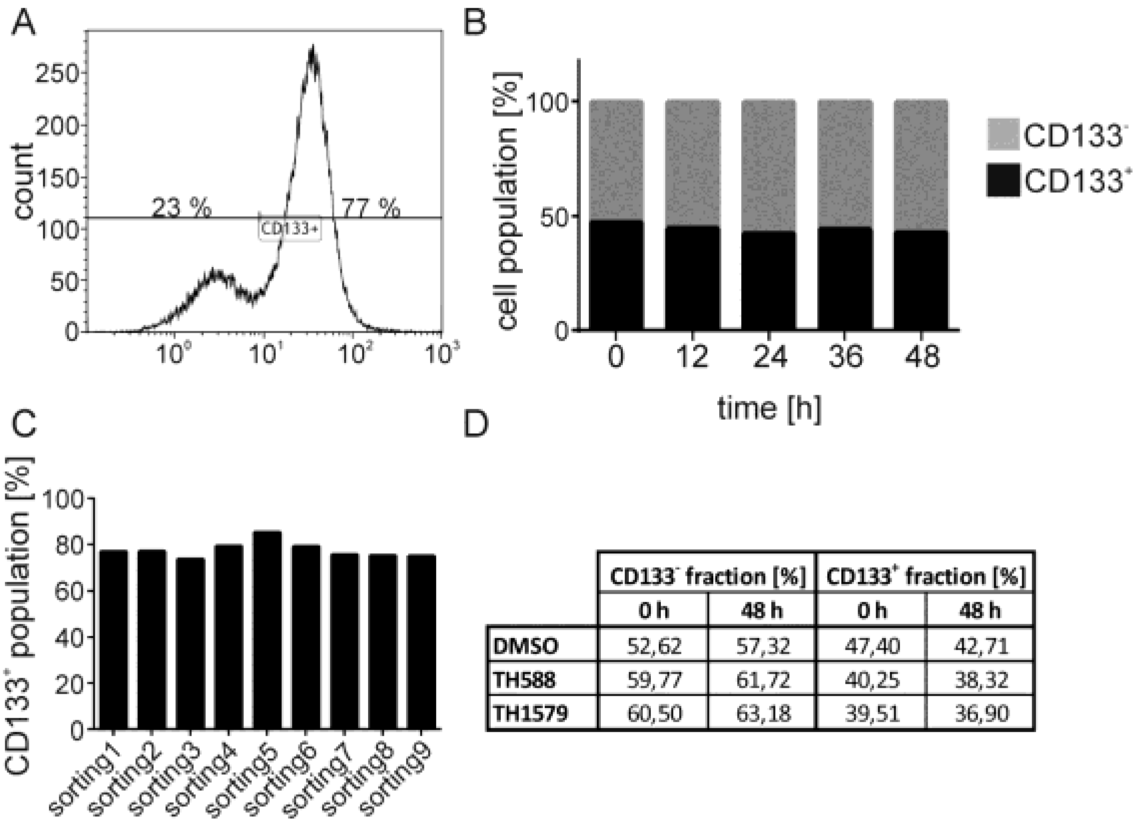

	CD133⁻ fraction [%]		CD133⁺ fraction [%]	
	**0 h**	**48 h**	**0 h**	**48 h**
**DMSO**	52,62	57,32	47,40	42,71
**TH588**	59,77	61,72	40,25	38,32
**TH1579**	60,50	63,18	39,51	36,90

Supplementary Figure 9:

Typical FACS plot of GMB line #18 cells after MACS sort for CD133 (A). MACS sorting is of consistent quality in between different experiments (B). The $CD133^+$ population is not lost due to spontaneous differentiation during the experimental time frame (C). The fraction of $CD133^+$ cells remains constant upon exposure to the MTH1 inhibitors TH588 and TH1579 (D)

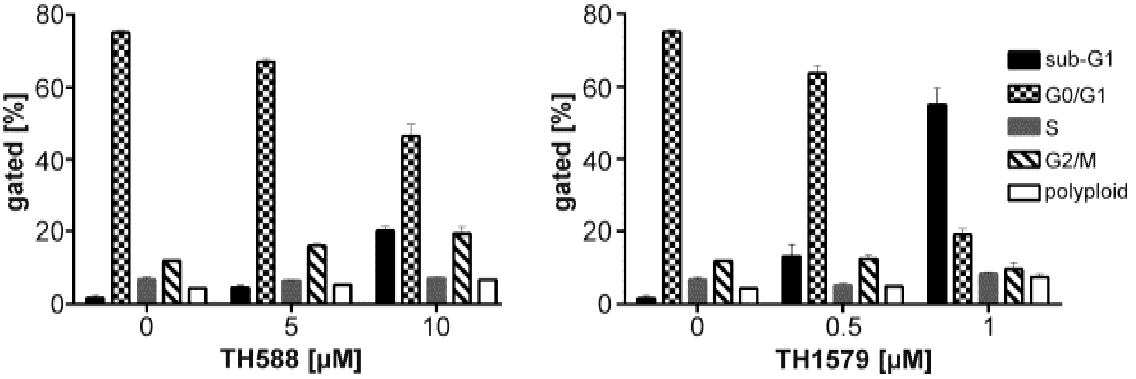

Supplementary Figure S10: Quantitation of cell cycle analysis of the CD133^{+} fraction

CD133^{+} cells have been isolated from GBM line #18 and treated with MTH1 inhibitors TH588 and TH1579 for 72 hours before cell cycle analysis.

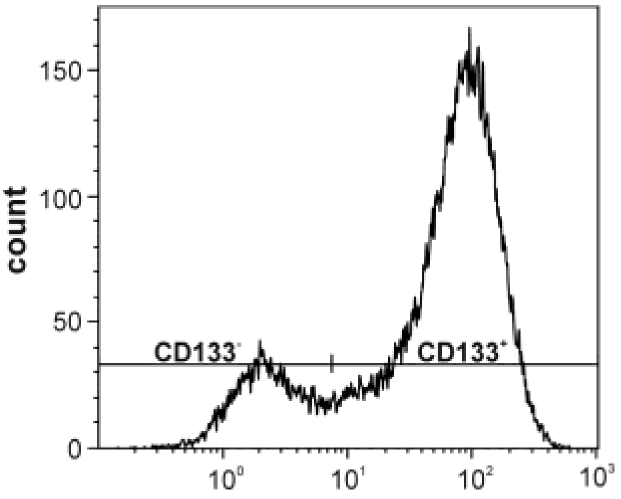

Supplementary Figure S11: FACS plot of xenotransplanted GBM cells

FACS plot of GBM#18:CMV-LUC cells with enriched CD133+ fraction before transplantation.

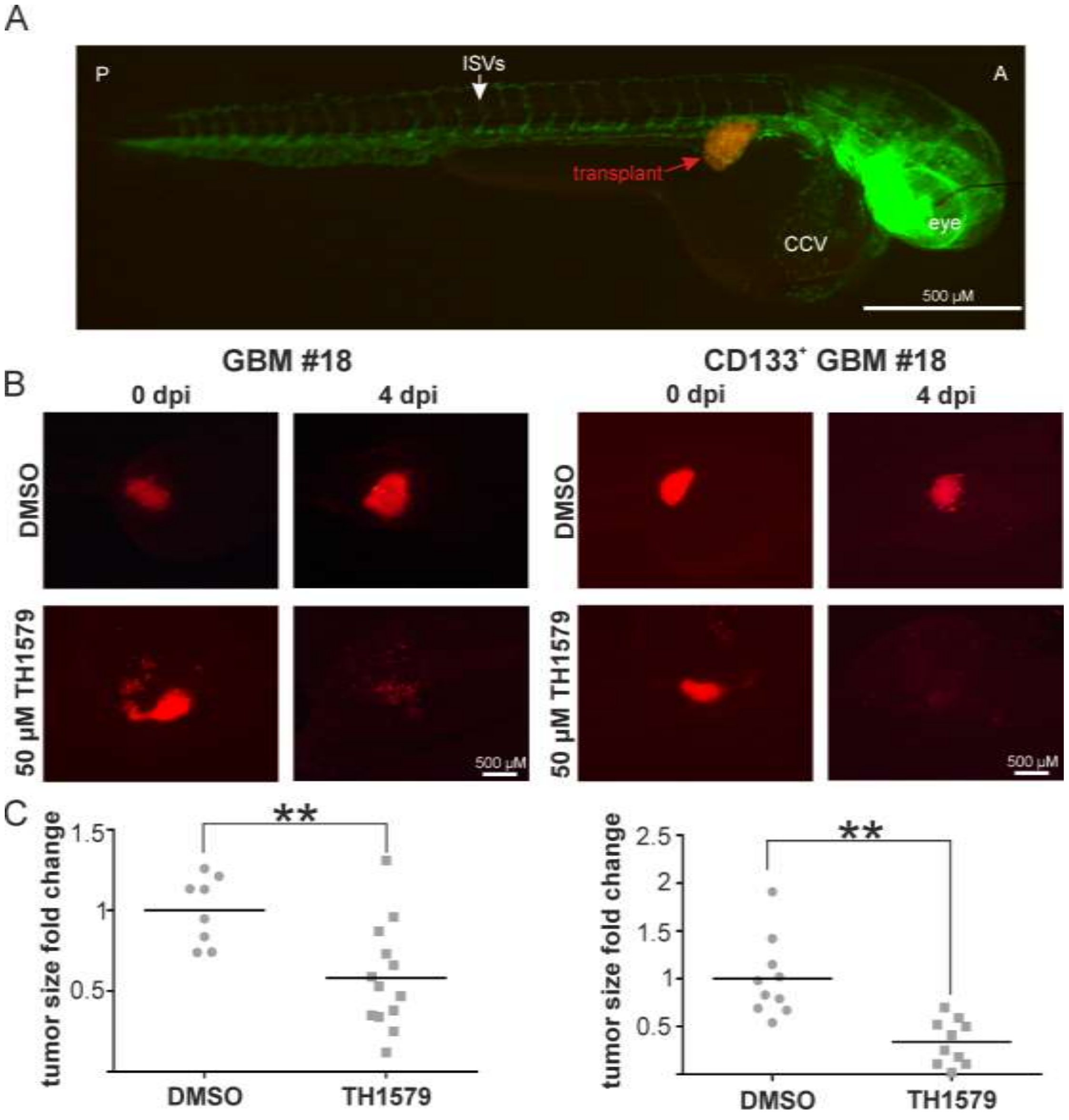

Supplementary Figure S12: non-orthotopic xenotransplantation

GBM cells were injected into the perivitelline space of two day old zebrafish embryos (A), which were subsequently exposed to 50 µM TH1579. The fold change of the tumor area was quantified 4 days after transplantation (B,C). Fold change upon TH1579 exposure for #18: 0.58 ± 0.32 compared to DMSO control, n = 13; p = 0.002 and for CD133^{+}: 0.34 ± 0.24 compared to DMSO control; n = 11; p = 0.005). Dpi: days post injection

Supplementary Movie S1: Real-time light sheet microscopy of orthotopic tumor

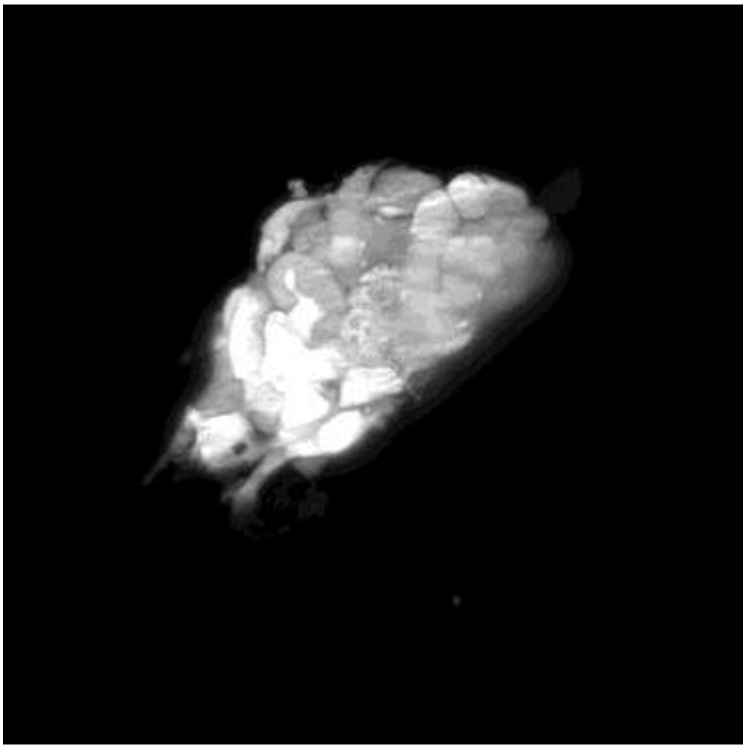

U343-MGA-GFP cells were transplanted orthotopically into the embryonic zebrafish brain, treated with 50 µM TH1579 and followed for 48 hours by real-time light sheet microscopy.
